# Treatment Options of Prosthetic Joint Infections Following Total Ankle Arthroplasty: A Systematic Review

**DOI:** 10.3390/jcm14030718

**Published:** 2025-01-23

**Authors:** Giacomo Capece, Emidio Di Gialleonardo, Chiara Comisi, Guido Bocchino, Virginia Cinelli, Antonio Mascio, Camillo Fulchignoni, Tommaso Greco, Giulio Maccauro, Carlo Perisano

**Affiliations:** 1 Orthopaedics and Trauma Surgery Unit, Catholic University of The Sacred Heart, 00168 Rome, Italy; emidiodiggia@gmail.com (E.D.G.); chiara.comisi22@gmail.com (C.C.); guido.bocchino@hotmail.it (G.B.); virginiacinelli23@gmail.com (V.C.); antonio.mascio87@gmail.com (A.M.); camillo.fulchignoni@gmail.com (C.F.); greco.tommaso@outlook.it (T.G.); giulio.maccauro@unicatt.it (G.M.); carlo.perisano@policlinicogemelli.it (C.P.); 2 U.O.C. Orthopedics and Traumatology, Ospedale dei Pellegrini, 80134 Naples, Italy; 3 Department of Ageing, Neurosciences, Head-Neck and Orthopaedics Sciences, Orthopaedics and Trauma Surgery Unit, Fondazione Policlinico Universitario Agostino Gemelli, IRCCS, 00168 Rome, Italy; 4 Department of Life Sciences, Health, and Health Professions, Link Campus University, 00165 Rome, Italy

**Keywords:** infected, ankle, arthroplasty

## Abstract

**Background**: This comprehensive systematic review aims to explore and discuss existing treatment modalities for infections in total ankle arthroplasty (TAA), providing insights that may contribute to the establishment of a “standard of care” for these challenging cases. The study analyses the intricate landscape of infected TAA, addressing gaps in the current literature and emphasizing the need to refine treatment strategies. With the reported incidence of periprosthetic joint infection after TAA surpassing rates observed in total hip and knee replacements, the research navigates through various treatment modalities, underscoring the lack of a universally accepted standard of care. **Methods**: In this systematic review, following PRISMA guidelines, PubMed, Scopus, and Google Scholar, we identified 15 papers addressing the management strategies for infected TAA (162 infected ankle arthroplasty cases).These databases were chosen for their extensive coverage, strong relevance to the research topic, and ease of access, ensuring a thorough and focused retrieval of pertinent literature on the treatment of infected ankle prostheses. The review involved the identification and evaluation of articles providing insights into complications, treatment outcomes, and risk factors. Extracted data were summarized and reported. A descriptive analysis was performed, and when feasible, a statistical analysis was conducted. **Results**: Treatment modalities included irrigation and debridement (48.8%), revision total ankle arthroplasty (36.3%), primary arthrodesis (7.9%), spacer arthroplasty (4.5%), and primary amputation (3.9%). Complication rates varied, with 46.5% for irrigation and debridement, 20% for two-stage revision, 7.14% for primary arthrodesis, and 25% for spacer arthroplasty. **Conclusions**: The rising prevalence of TAA underscores the need for a definitive treatment protocol due to severe complications. This review emphasizes careful patient selection and accurate diagnosis. Irrigation and debridement are effective for acute infections, while two-stage revision is a valid alternative for chronic infections. High-quality randomized controlled trials are important for establishing an evidence-based treatment protocol.

## 1. Introduction

### 1.1. Background

End-stage ankle osteoarthritis, causing substantial pain and loss of function, is traditionally found treatment in ankle arthrodesis (AA). Despite successful outcomes, the emergence of third-generation implants has popularized total ankle arthroplasty (TAA) as an effective alternative, preserving motion and challenging the longstanding acceptance of arthrodesis.

A significant and potentially devastating complication associated with TAA is periprosthetic joint infection (PJI), with significant morbidity and substantial healthcare costs. The management often involves surgical treatments such as debridement, two-stage revision surgery with implantation of a cement spacer, conversion to ankle arthrodesis, or, in extreme cases, amputation [1]. The reported incidence of PJI for TAA varies between 1.2% and 8.6%, exceeding the rates observed in total hip (THA) and knee (TKA) replacements, which are 1.9% (ranging from 1.5% to 2.2%) following TKA and 1.5% (ranging from 1.3% to 1.7%) following THA, respectively [2]. Despite TAA’s recent popularity, the optimal treatment for PJI in this context remains undefined. Limited literature exists on the management of infected TAA and the applicability of algorithms developed for hip and knee PJI for the ankle is also unclear [3]. A significant gap in the current literature is the lack of standardized treatment protocols specifically for TAA-associated infections, particularly when compared to the more well-defined protocols for hip and knee arthroplasties. This review aims to address this gap by analyzing the existing treatment modalities and evaluating their effectiveness in managing PJI after TAA.

### 1.2. Risk Factors

The soft tissue envelope of the ankle joint presents a challenge in managing infections post-TAA. Identifying preoperative patient characteristics correlating with an increased risk of PJI is crucial for appropriate patient selection. Risk factors for prosthetic ankle infection include patient characteristics for example inflammatory arthritis, prior ankle surgery, age less than 65 years, body mass index less than 19, peripheral vascular disease, chronic lung disease, hypothyroidism, and low preoperative American Orthopaedic Foot and Ankle Society (AOFAS) hindfoot scores [4,5,6]. There is discordance in the literature regarding the role of risk factors such as obesity, tobacco use, diabetes, and duration of surgery on the occurrence of infections of ankle prosthesis [7]. Given the morbidity associated with infected total ankle replacement, careful consideration should be given to performing these procedures in patients with multiple prior surgeries and comorbidities predisposing them to wound healing difficulties [7].

### 1.3. Signs and Symptoms and Diagnostic Methods

The clinical presentation of periprosthetic ankle joint infection can exhibit significant variability: early acute and late acute versus chronic infections. Early acute infections arise within the first thirty days and late acute postoperative infections typically arise within the first 3 months following the procedure [8], characterized by a sudden onset of pain, local indicators of infection such as erythema, cellulitis, drainage, and systemic symptoms like fever, chills or night sweats. Conversely, chronic infections predominantly emerge beyond this initial period. These delayed infections might stem from indolent organisms necessitating extended incubation periods to provoke clinical infection or from hematogenous spread originating from dental, genitourinary, or gastrointestinal procedures. Patients exhibiting clinical symptoms and signs indicative of periprosthetic ankle infection, such as pain, erythema, warmth, sinus tracts, or abscesses around the wound alongside sinus tracts communicating with the ankle/subtalar joint are likely to be suffering from TAA infection [9]. The initial assessment of individuals suspected of periprosthetic joint infections should encompass blood tests measuring C-reactive protein and erythrocyte sedimentation rate. Joint aspiration and analysis of synovial fluid can further aid in confirming suspected periprosthetic ankle infection [10].

### 1.4. Treatment Options

Several treatment options for ankle PJI exist ranging from long-term suppressive antibiotic treatment to more invasive procedures such as debridement and implant retention (DAIR), 1-stage, 2-stage, and 3-stagerevision surgery, prosthesis component removal with cement spacer implantation, arthrodesis, and amputation [11].

Debridement, antibiotic, and implant retention (DAIR) involve aggressive cleansing and joint lavage with removal of scar tissue, obtaining multiple cultures for examination, replacing mobile components, and initiating empirical antibiotic therapy pending specific treatment based on the antibiogram obtained from peri-prosthetic biofilm analysis [12]. Revision total ankle arthroplasty represents a commonly adopted treatment strategy, which is divided into 1-stage and 2-stage revisions. In 1-stage revision, infected prosthetic components are removed, extensive debridement is performed, and, in the absence of signs of infection, a new revision prosthesis is implanted during the same surgical procedure. In the 2-stage revision, infected prosthetic components are removed, debridement is performed, and an antibiotic spacer is implanted; the new revision prosthesis is then implanted in a subsequent surgical procedure [13]. In 3-stage revision, the antibiotic spacer is replaced with another antibiotic spacer in a second surgical procedure, and the definitive revision prosthesis is implanted during the third surgical procedure. Another treatment strategy is primary arthrodesis [14]. This technique involves the complete removal of prosthesis components and definitive conversion to tibiotalar arthrodesis [15,16,17].

### 1.5. Study Objectives

This systematic review aims to identify and discuss medical and surgical treatment options for TAA infections in order to define a “standard of care.” Our systematic review compares the clinical effectiveness of various treatment strategies for PJI after TAA, emphasizing infection control as a primary outcome, while secondary includes measures of pain, function, satisfaction, non-infection-related adverse events, conversion to arthrodesis, and amputation.

## 2. Materials and Methods

The review adhered to the PRISMA (Preferred Reporting Items for Systematic Reviews and Meta-Analyses) guidelines [18], ensuring a comprehensive and systematic approach to data retrieval and synthesis. This systematic review has been appropriately registered with the International Prospective Register of Systematic Reviews (PROSPERO) under registration number 526324.

### 2.1. Search Strategy

The research was carried out on three electronic databases (PubMed, Scopus, and Google Scholars) by two authors independently (GC and EDG) from 1 April 2023 up to 12 March 2024, utilizing a combination of keywords and text terms such as “Infected total ankle arthroplasty”, “Periprosthetic ankle infection”, and “Revision ankle replacement”. The exact search string used was ((ankle infection [Title/Abstract]) OR (revision [Title/Abstract]) AND (ankle [Title/Abstract])). The eligibility criteria for inclusion in our review were established to ensure the selection of studies meeting rigorous standards. Studies were included if they were designed as case reports, case series, or randomized controlled trials (RCTs) and focused on the surgical treatment options of ankle PJI.

Exclusion criteria encompassed studies designed as systematic reviews, meta-analyses, and experimental studies (including in vitro studies, animal studies, or cadaveric studies). Additionally, the literature references of identified papers were analyzed to discover further relevant articles, with consideration given to all journals.To minimize the risk of missing studies, no time restrictions were applied to the search strategies. Article titles and abstracts underwent review and those of interest were selected for full-text examination. The bibliography of selected studies was meticulously searched by hand to identify additional studies not discovered in the electronic search. There were no restrictions on the date of publication or language.

The databases PubMed, Scopus, and Google Scholar were chosen for their comprehensive coverage, relevance to the research topic, and ease of access, ensuring a thorough and focused retrieval of pertinent literature. Although databases like Embase and the Cochrane Library were considered, preliminary searches on these platforms yielded a limited number of studies that met our inclusion criteria, which were already captured by the selected databases. This approach helped maintain the focus and relevance of the search while ensuring methodological consistency.

After conducting the literature search and applying filters to exclude systematic reviews, meta-analyses, and experimental studies, 58 articles were identified from PubMed. A similar search in Google Scholar yielded 519 results, filtered exclusively by scientific articles. These results were then manually screened to identify relevant studies, ultimately resulting in 24 articles suitable for evaluation after removing duplicates already identified in PubMed. The search in Scopus identified 64 articles, of which 6 unique scientific articles remained after excluding duplicates found in PubMed.

**Figure 1:**
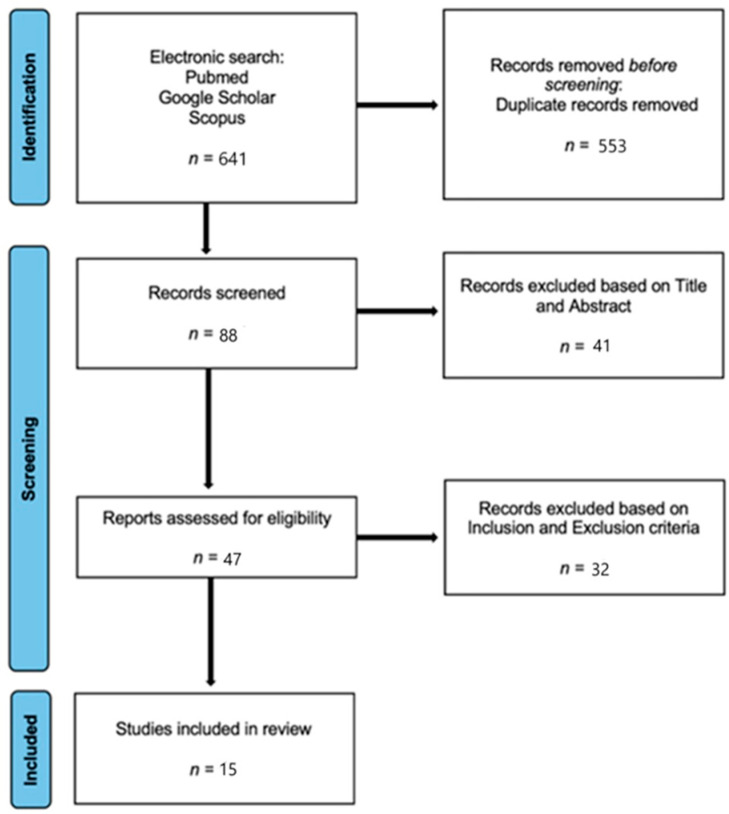
PRISMA flow-chart. Flow diagram illustrating the study selection process, including the number of records identified, screened, excluded, and included in the final analysis.

In total, 88 papers were selected for further evaluation (PRISMA Flowchart, Figure 1). Of these, 41 were initially excluded based on the title or abstract: 39 articles did not address total ankle arthroplasty (TAA) revisions but instead focused on prosthesis revisions in other areas or treatments for fractures or other foot and ankle traumas; 1article discussed the use of a surgical helmet system during TAA surgery, and another addressed the preparation of the surgical field during such procedures. Subsequently, 32 papers were excluded as they focused on aseptic revisions of TAA or septic revisions of ankle arthrodesis. Finally, 15 papers (published between 2009 and 2023) met the inclusion criteria. Among these studies, 71.4% were randomized clinical trials, and 5 were case series.

Two reviewers (GC and EDG) assessed the full text of the selected articles to determine their eligibility for inclusion and gathered data of interest. In cases of uncertainty regarding the inclusion of an article, the senior author made the final decision.

The two authors (GC and EDG) independently assessed the risk of bias. A supervisor [CP] was consulted in case of disagreement. Titles of journals, names of authors, and supporting institutions were not masked at any stage. No attempt was made to contact authors to obtain individual patient data. In case of disagreements between the two reviewers (GC and EDG) regarding the inclusion of articles, the senior author (CP) was consulted to resolve the issue.

To assess the quality of the studies, the Coleman Methodology Score (CMS) was used, which assesses methodology with 10 criteria, giving a total score between 0 and 100. A score of 100 indicates that the study largely avoids chance, various biases, and confounding factors. The subsections that make up the CMS are based on the subsections of the Consolidated Standards of Reporting Trials (CONSORT) statement (for randomized controlled trials) and are modified to allow for other trial designs.

The Coleman criteria were modified to make them reproducible and relevant for the systematic review of treatment options for prosthetic joint infections following total ankle arthroplasty. Each study was scored by 2 reviewers (G.C., E.D.G.) independently.

### 2.2. Data Extraction and Analysis

Detailed information was systematically extracted from each selected study. The extracted information included the number and gender of participants, the type of prosthesis used, the pathogens isolated that caused the prosthesis infection, the type of treatment administered, and the assessment of long-term treatment efficacy.

Statistical analysis was performed using SPSS 18.0 for Windows (SPSS Inc., Chicago, IL, USA). Descriptive statistics were used to summarize the findings across all the included studies.

## 3. Results

### 3.1. Demographics

In total, data from 162 patients who developed infection after TAA were analyzed. In two studies, the gender of the enrolled patients remains unspecified, accounting for a total of 13 patients, from the remaining studies, 87 (58.3%) were male and 62 (41.6%) females. Regarding the age of the enrolled patients, it is specified in all the studies analyzed except for one [19], with an average age of the enrolled patients of 54.51 years old (range 23–65) [Table 1].

In the analysis of the prostheses used across the studies, it is noted that in 8 out of the 15 articles (53.3%), the specific type of prosthesis implanted was not reported.

For the articles that did specify the type of prosthesis, the INBONE prosthesis (Stryker, Mahwah, NJ, USA) was used in 17 cases (10.5%), the Salto-Talaris Total Ankle Prosthesis (Tornier SA, Saint Ismier, France) appeared in 12 cases (7.4%), the STAR prosthesis (Enovis, Wilmington, DE, USA) was utilized in 5 cases (3.1%), the Vantage prosthesis (Exactech, Gainesville, FL, USA) was chosen in 2 cases (1.2%), the Agility Total Ankle System (DePuy Orthopedics, Wausau, IN, USA) was the most frequently used, with 36 cases (22.2%). Lastly, the Invision prosthesis (Stryker Way Portage, Portage, MI, USA) was implemented in 5 cases (3.1%).

In 11 scientific articles (64.7%) within our search scope, predisposing risk factors for infection are examined. These factors often entail specific comorbidities among individual patients, which are frequently shared. Delving into the details of predisposing pathologies, we observed 21 patients (11.9%) afflicted with unspecified cardiovascular diseases, 33 patients (18.7%) with uncompensated diabetes under treatment, 27 patients (15.3%) who were smokers overall, including 6 who had recently ceased tobacco use. Additionally, one patient was diagnosed with sleep apnea, twelvepatients (6.8%) with rheumatoid arthritis, one with arthrogryposis, and finally, fourpatients (2.2%) with hepatitis C [Table 2]. Many patients examined in our systematic review had undergone previous ankle surgeries, which can be considered a risk factor for developing infectious complications during definitive ankle prosthesis treatment. Specifically, 19 patients (10.7%) had undergone initial total ankle prosthesis implantation, 10 patients (5.6%) had undergone initial arthrodesis that was subsequently converted to ankle prosthesis, and 2patients (1.1%) had previously undergone surgical treatment for tibial Pilon fractures using synthesis methods [Table 2].

### 3.2. Diagnosis and Type of Infection

In 90% of the scientific articles analyzed, a diagnosis of deep infection was made, while the remaining 10% reported superficial infection. Specifically analyzing the timing of infection onset, it was found that in three(20%) of the scientific articles, this was not specified. Early acute infections develop in 12.96% of cases (21 patients), late acute infections in 24% of cases (39 patients), and chronic infections in 39.5% of cases (64 patients) [Table 3].

Our scientific analysis has led us to identify 120 isolated species through microbiological samples taken from biopsies or synovial fluid near the components of the ankle prosthesis. Additionally, in 46.6% of the cases, the explanted prosthetic components were sent for sonication. In three scientific studies (18.7%), the isolated pathogens that caused prosthesis infection are not described. Out of 95 identified species (79.1%), *Staphylococcus aureus* was found. Specifically, 14 samples (14.7%) tested positive for *methicillin-resistant Staphylococcus aureus* (MRSA), 30 (31.5%) tested positive for *methicillin-sensitive Staphylococcus aureus* (MSSA), and 15 (15.7%) for *coagulase-negative staphylococci (CoNS)*. Additional identified species include *Escherichia coli* (2.5%), *Streptococcus viridans* (2.5%), *Streptococcus caprae* (2.5%), *Staphylococcus epidermidis* (2.5%), *Pseudomonas aeruginosa* (2.5%), and *Staphylococcus lugdunensis* (3.3%). In a minority (0.8%), additional pathogens such as *Enterobacter* spp., *Propionibacterium* spp., *Corynebacterium* spp., and *Streptococcus mitis* were identified [Table 4].

### 3.3. Treatment Options and Outcomes

Surgical treatments were reported in all studies, and the different types of treatments are listed in Table 5 and described in the following paragraphs. Overall, our study revealed that the most frequently performed surgical treatment was DAIR, which was applied to 71 patients (44.7%).

Focusing on clinical treatment with antibiotic therapy, this was described in seven studies. Specifically, in the studies by Short et al. [23], Miller et al. [26], Mulhern et al. [27], Reuver et al. [19], Young et al. [29], and Kliushin et al. [31], antibiotic therapy was reported as part of a medical approach combined with an antibiotic spacer. In the studies by Short et al. [23], Mulhern et al. [27], McCoy Jr. et al. [28], Reuver et al. [19], and Patton et al. [30], the specific antibiotic used was not mentioned. In Miller’s study [26], vancomycin was used as the antibiotic therapy. In the study by Young et al. [29], the combination of vancomycin and piperacillin/tazobactam was used initially, followed by ceftriaxone, and finally penicillin G. In the study by Kliushin et al. [31], Rifampin and Augmentin were used as the antibiotics.

#### 3.3.1. Irrigation, Debridement, and Polyethylene Exchange

Upon specific analysis of various treatment modalities, irrigation and debridement (I&D) emerge as a primary strategy, employed in 72 patients (48.8%), which is widely used in managing infectious complications. This approach is typically coupled with polyethylene insert replacement and a six-week course of intravenous antibiotic therapy. Widely recognized as debridement, antibiotic, and implant retention (DAIR), this procedure was initially developed as the primary approach for managing infectious complications of knee and hip prostheses but has also demonstrated effectiveness in ankle prosthesis infections. However, the high rate of complications in DAIR, nearly reaching 50%, raises doubts about its appropriateness for post-TAA infections.

The DAIR procedure involves thorough cleansing and lavage of the joint, removal of scar tissue, multiple cultures (preferably after a minimum of 15 days without antibiotic therapy), replacement of mobile components, and initiation of empirical antibiotic therapy pending specific treatment based on peri-prosthetic biofilm analysis. Commonly used antibiotics include vancomycin and ceftriaxone, adjusted according to the sensitivity of the identified pathogen. This technique’s advantage lies in its rapid execution without the need for removal of fixed prosthesis components. However, its effectiveness varies in the literature, particularly in late-stage infections, with conflicting results. When performed within the appropriate timeframe, it shows high success rates, supported by the fact that within the first 3–4 weeks, the biofilm is still reversible. Among the 72 patients treated with DAIR in our review, 33 patients (46.5%) experienced a procedure failure, possibly due to pathogen factors, patient clinical conditions, or delayed execution. Specifically, in the study by Kessler et al. [20], out of 21 patients treated with DAIR, 4 (19%) experienced recurrence. The sixpatients treated with DAIR in the study by Anastasio et al. [21] had no complications and achieved a 100% healing rate. In the study by Pfahl et al. [22], out of sevenpatients treated with DAIR, three(42%) experienced recurrence. Similar findings were reported in studies by Myerson et al. [24] and Lachman et al. [25], where complications were observed in 84% and 50% of patients, respectively [Table 6].

#### 3.3.2. Revision Total Ankle Arthroplasty

In 64 cases (36.3%) analyzed in our search string, total revision of the ankle arthroplasty was adopted as the procedure, specifically described in three different treatment types: one-stage, two-stage, and three-stage. The one-stage revision technique was adopted in twopatients (1.13%) and showed a failure rate of 0%, likely attributed to the small sample size. It is a fairly demanding technique involving complete removal of all components (fixed and mobile) of the infected prosthesis, thorough debridement of the joint cavity, and immediate implantation of a new revision prosthesis. This procedure is often accompanied by a course of intravenous antibiotics for 6–12 weeks, typically involving broad-spectrum antibiotics like vancomycin and piperacillin–tazobactam, tailored to the identified pathogen’s sensitivity. It represents a valid surgical option in cases of delayed or chronic peri-prosthetic infection where the causative pathogen has been isolated and is susceptible to antibiotics, the patient is not at risk and has undergone only one previous surgical intervention. Pfahl et al. [22] and Patton et al. [30] analyzed this one-stage strategy in one patient each. On the other hand, the two-stage complete revision is a more widely used option, adopted in 60 patients (34%) in our search string, with a failure rate of 20% (12 patients). This type of revision constitutes the most valid and secure option in cases of peri-prosthetic infection difficult to treat due to reasons related to resistant or difficult-to-isolate pathogens, as well as reconstructive difficulties due to significant bone and/or soft tissue loss. The two-stage surgical process involves the initial removal of the infected prosthesis, extensive debridement, and placement of an antibiotic-laden spacer, followed by a period of intravenous antibiotics, typically vancomycin combined with cefepime or meropenem, for 6–12 weeks. This is followed by re-implantation of a new prosthesis once the infection has cleared. The drawbacks of this technique include the need for two surgical interventions and prolonged times for definitive healing. Specifically, the two-stage technique was adopted in 10 patients in the study by Kessler et al. [20] with a 100% success rate, in 7 patients in the study by Anastasio et al. [21] with a 100% success rate, as well as in other studies such as those by Pfahl et al. [22], Miller et al. [26], Mulhern et al. [27], McCoy Jr. et al. [28], Young et al. [29], Patton et al. [30], and Kliushin et al. [31], all showing a 100% success rate following this technique. In one patient in the study by Short et al. [23], there was a persistence of surgical wound dehiscence, requiring additional irrigation and debridement with associated orthoplastic surgery. Among the elevenpatients treated with the two-stage technique in the scientific study by Conti et al. [32], complications developed in 54% of cases: infection persisted in threepatients, prosthetic component mobilization occurred in one patient, and deformity remained in twopatients. The three-stage prosthesis revision technique was adopted in twopatients (1.13%) and showed a failure rate of 50%. This involves a scheduled spacer exchange and prolonged antibiotic treatment, typically 12–16 weeks of antibiotics tailored to the pathogen, involving agents like vancomycin and meropenem. This technique is particularly described in the study by Pfahl et al. [22], where a three-stage revision (oneto arthrodesis, one to arthroplasty) was performed [Table 6].

#### 3.3.3. Primary Arthrodesis

Another revision technique described in 14 patients (7.9%) is primary arthrodesis, associated with a 7.14% complication rate. The technique involves the complete removal of prosthesis components and definitive conversion to tibiotalar arthrodesis. The advantage of this technique lies in its greater simplicity compared to prosthetic re-implantation; however, it results in the complete elimination of joint motion, albeit relieving the patient of pain. Antibiotic therapy typically includes a 6–8-week course of intravenous antibiotics, such as vancomycin and piperacillin–tazobactam, adjusted according to the pathogen’s sensitivity. Nevertheless, it entails the adoption of additional fixation means that may promote bacterial biofilm growth, and delayed consolidation or pseudoarthrosis may occur, as seen in a patient treated in the study by Patton et al. [30]. Regarding the risk of fixation device superinfection, the possibility of performing arthrodesis with an external fixator such as the Ilizarov type [31] should also be considered, which eliminates this disadvantage. This technique is usually adopted as an end-stage procedure in ankle prosthesis failure, and generally, in 90% of cases, it results in complete healing without recurrence, improving the patient’s quality of life [Table 6].

#### 3.3.4. Spacer Arthroplasty and Primary Amputation

Additional procedures reserved for the failure of other treatment strategies for ankle prosthesis infections include definitive treatment with antibiotic spacers, used in eightpatients (4.5%), and primary below-knee amputation (BKA) performed in sevenpatients (3.9%). In the studies by Short et al. [23], Ferrao et al. [33], and Kliushin et al. [31], antibiotic spacers with gentamicin and vancomycin were implanted. In the study by Miller et al. [26], an antibiotic spacer with vancomycin and tobramycin was used. In the studies by Mulhern et al. [27] and Young et al. [29], spacers with only gentamicin were placed. In twopatients from the study by Patton et al. [30] and in all patients from the study by Ferrao et al. [33], treatment with articulated antibiotic spacers was performed, allowing for a limited range of ankle motion and weight-bearing on that limb. However, in 25% of patients treated with definitive antibiotic spacers, failure occurred due to spacer rupture and persistent infection, leading to the last resort treatment, below-knee amputation [Table 6].

## 4. Discussion

TAA has become an increasingly popular procedure for managing end-stage ankle arthritis [34,35]. However, the rise in its prevalence has been accompanied by a surge in severe complications, particularly prosthetic joint infections, imposing substantial morbidity and financial burdens on healthcare systems [19]. The reported incidence of periprosthetic joint infection after TAA varies widely in the literature, ranging from 1.2 to 8.6 percent [36], surpassing rates observed in total hip and knee replacements. Despite the growing adoption of TAA, a definitive and universally accepted treatment protocol for prosthetic joint infections in this context remains elusive. Gaps in the literature highlight the need for further research to refine and optimize treatment strategies [37].

The management of infected total ankle replacements is an understudied domain, characterized by variable surgical outcomes and elevated complication rates. Additionally, the applicability of algorithms developed for hip and knee arthroplasty infections to the unique challenges presented by infected TAA remains unclear. Risk factors for prosthetic ankle infections span patient characteristics, surgical considerations [38], and the postoperative course, emphasizing the need for meticulous patient selection. Given the heightened morbidity associated with infected total ankle replacements, special attention must be given to patients with a history of multiple prior surgeries and comorbidities that predispose them to complications related to wound healing.

Our study presents a thorough investigation into the complexities surrounding complications, treatment modalities, and outcomes associated with total ankle arthroplasty (TAA) management. The insights gleaned from our review offer valuable guidance for both patients and healthcare practitioners engaged in the management of ankle prosthesis-related complications. Our findings resonate with those of previous research, reinforcing the multifaceted nature of ankle prosthesis infections.

As noted by Walley et al. [4], understanding the demographics of patients experiencing post-surgical infections is crucial for tailoring effective treatment strategies. In our review of 15 scientific studies encompassing 162 patients over a 15-year period, we observed a predominant middle-aged cohort, with an average age of 54.51 years.

The identification of predisposing risk factors for infection aligns with the observations made by Da Silva et al. [39] regarding the significance of comorbidities in prosthetic joint infections. Consistent with their findings, our analysis revealed common comorbidities such as cardiovascular diseases, diabetes, and a history of smoking among patients who developed ankle prosthesis infections.

Microbiological analysis played a pivotal role in elucidating the diverse range of pathogens implicated in ankle prosthesis infections. Our review underscores the prevalence of *Staphylococcus Aureus*, including *Methicillin-Resistant Staphylococcus Aureus (MRSA)* and *Methicillin-Sensitive Staphylococcus Aureus (MSSA)*, as predominant causative agents. This highlights the importance of tailored treatment approaches based on individual patient profiles and microbiological findings.

The treatment landscape for ankle prosthesis infections is multifaceted, as evidenced by the varied therapeutic modalities identified in our review. The efficacy of irrigation and debridement (I&D), as discussed by Uçkay et al. [12], remains subject to debate, with our analysis revealing variable success rates across different studies. Notably, the variability in outcomes can be attributed to factors such as the presence of biofilm, delayed infection diagnosis, and inadequate targeted antibiotic therapy. Addressing these factors through early detection, microbiological assessment, and tailored treatment strategies could improve the success rates of I&D, particularly in acute cases. Future research should also focus on specific targeted antibiotic therapy; indeed, as highlighted by Parekh et al. [40], culture-directed antibiotic therapy is recommended for patients undergoing operative treatment of infected total ankle arthroplasty (TAA). Routine administration of suppressive antibiotics in patients with an ankle prosthesis in place is not warranted; however, in certain clinical circumstances, this may be of benefit.

Special consideration should also be given to patients with chronic infections, as they present unique challenges. These patients may require more intensive treatment strategies, including prolonged antibiotic regimens and potentially more invasive surgical approaches such as two-stage revision, given the difficulty of eradicating infection in the presence of biofilm and tissue damage [17].

Similarly, the merits of revision total ankle arthroplasty, including one-stage, two-stage, and three-stage techniques, have been extensively deliberated in the literature. Our review concurs with the findings of Lu et al. [41] regarding the efficacy of two-stage revision as a widely adopted and secure option for managing challenging ankle prosthesis infections.

A strategy to be adopted and focused on for future research could be the utilization of closed incision negative pressure therapy immediately after total ankle arthroplasty surgeries; this idea, introduced by Sidorski et al. [42], could greatly reduce the chances of surgical wound dehiscence and infectious complications.

In summary, our comprehensive analysis sheds light on the intricate interplay between patient demographics, risk factors, pathogens, and treatment outcomes in the management of ankle prosthesis-related complications. By synthesizing findings from various studies, we provide valuable insights that can inform evidence-based decision-making and optimize patient care in clinical practice. Future research should focus on addressing these complexities to improve overall patient outcomes in TAA and its complications. Key takeaways include the need for refined interventions and standardized diagnostic approaches. The collective insights derived from these studies enrich our understanding of postoperative infections in TAA, guiding future research trajectories and enhancing clinical decision-making in this intricate domain.

### Study Limitations

This review is subject to several limitations that warrant consideration. A primary constraint is the restricted number of included studies, especially in the assessment of outcomes, treatment types, and results during follow-up. Additionally, the retrospective nature of the study inherently limits the ability to draw definitive causal conclusions due to potential biases in data collection and reporting.

The research was confined to only three electronic databases, namely PubMed, Scopus, and Google Scholar. While these are commonly utilized sources, there are additional databases that could ensure a more comprehensive coverage of the literature, such as Embase or the Cochrane Library. The scarcity of data on applied treatments and the diverse outcome measurement scales employed by various authors pose challenges in accurately assessing and comparing outcomes. Moreover, some studies included in the review lacked detailed data on antibiotic regimens, which are crucial for understanding the complete treatment context. The variation in surgical techniques and postoperative care further complicates the comparison across different studies. To comprehensively understand the long-term effects of distinct revision surgery strategies, there is a crucial need for extended follow-up studies. It is important to acknowledge that our focus was solely on studies related to infections in total ankle arthroplasty (TAA), and the management of infections following arthrodesis was not considered. Future research should encompass a broader scope to provide a holistic understanding of ankle infection management strategies. Despite these limitations, our systematic examination of diverse TAA infection treatment strategies, backed by meticulous evaluation of primary and secondary outcomes, bolsters the reliability of our results. The consistently substantial magnitude of the associations observed in our study further underlines the significance and validity of these findings.

## 5. Conclusions

The discussion underscores the escalating incidence of severe complications accompanying the rising prevalence of TAA and underscores the necessity for a definitive treatment protocol. This review offers valuable insights for both patients and healthcare practitioners, emphasizing the importance of cautious patient selection, the formidable challenges posed by infections, and the criticality of precise diagnosis. Serving as a contribution to the current understanding of TAA, the study lays the groundwork for future research trajectories and facilitates enhanced clinical decision-making in this complex domain. While irrigation and debridement, along with two-stage revision, emerged as viable treatments for acute postoperative infections and two-stage revision represents viable treatment for late chronic infections, a discernible trend towards preserving articular function through two-stage revision was observed. However, it is imperative to acknowledge the weak quality of evidence, characterized by biases in reporting and selection processes. High-quality randomized controlled trials are imperative to compare different treatments and establish an evidence-based treatment protocol.

## Figures and Tables

**Table 1:** Demographic characteristics of the analyzed patients (M: male; F: female; CMS: Coleman Methodology Score).

RESEARCH STUDY	STUDY DESIGN	NUMBER OF PATIENTS	GENDER	MEAN AGE (Years)	TYPE OF PROSTHESIS	CMS
Kessler et al. (2014) [20]	Retrospective	34	20 F; 14 M	62.1	Not specified	42
Anastasio et al. (2023) [21]	Retrospective	19	5 F; 14 M	60.4	INBONE; Salto-Talaris; STAR; Vantage	47
Pfahl et al. (2022) [22]	Retrospective	20	7 F; 13 M	65	Not specified	40
Short et al. (2020) [23]	Case report	1	M	61	Not specified	22
Myerson et al. (2014) [24]	Retrospective	19	8 F, 11 M	65	Agility	32
Lachman et al. (2018) [25]	Retrospective	14	9 F, 5 M	60.9	Not specified	41
Miller et al. (2017) [26]	Case report	1	M	23	Not specified	50
Mulhern et al. (2016) [27]	Case report	1	M	56	INBONE	40
McCoy Jr. et al. (2012) [28]	Retrospective	3	1 F; 2 M	60	Not specified	36
Reuver et al. (2010) [19]	Retrospective	2	N/A	N/A	Salto Total Ankle Prosthesis	45
Young et al. (2009) [29]	Case report	1	M	58	Agility	39
Patton et al. (2012) [30]	Retrospective	29	12 F; 17 M	29	Salto-Talaris; Agility; INBONE; STAR	40
Kliushin et al. (2020) [31]	Case report	1	M	32	Not specified	23
Conti et al. (2022) [32]	Retrospective	11	N/A	61.18	INBONE; Invision	41
Ferrao et al. (2012) [33]	Retrospective	6	M	63.3	Not specified	43
	Total	162	62 F; 87 M; 13 N/A	54.51 (median 60.9)		

**Table 2:** Risk factors.

RISK FACTORS	NUMBER OF AFFECTED PATIENTS
Cardiovascular diseases	21 (11.9%)
Diabetes	33 (18.7%)
Smoking	27 (15.3%)
Sleep Apnea	1 (0.5%)
Rheumatoid Arthritis	12 (6.8%)
Arthrogryposis	1 (0.5%)
Hepatitis C	4 (2.2%)
Previous surgery	31 (17.6%)

**Table 3:** Type of infection and timing.

RESEARCH STUDY	TYPE OF INFECTION	TIMING
Kessler et al. (2014) [20]	Deep infection	Late acute 55.9% Chronic 44.1%
Anastasio et al. (2023) [21]	Deep infection	Not specified
Pfahl et al. (2022) [22]	Deep infection	Early acute 10% Late acute 20% Chronic 70%
Short et al. (2020) [23]	Deep infection	Early acute
Myerson et al. (2014) [24]	Deep infection	Early acute 5% Late acute 16% Chronic 79%
Lachman et al. (2018) [25]	Deep infection	Early acute 5% Late acute 16% Chronic 79%
Miller et al.(2017) [26]	Deep infection	Early acute
Mulhern et al. (2016) [27]	Deep infection	Chronic
McCoy Jr. et al. (2012) [28]	Superficial infection	Early acute
Reuver et al. (2010) [19]	50% Superficial infection 50% Deep infection	Not specified
Young et al. (2009) [29]	Deep infection	Early acute
Patton et al. (2012) [30]	Deep infection	Early acute 38% Late acute 38% Chronic 24%
Kliushin et al. (2020) [31]	Deep infection	Chronic
Conti et al. (2022) [32]	Deep infection	Not specified
Ferrao et al. (2012) [33]	Deep infection	Late acute: 44.4% Chronic: 22.2%

**Table 4:** Type of pathogen.

TYPE OF PATHOGEN	N° OF SAMPLES	FREQUENCY
*Methicillin-Resistant Staphylococcus aureus (MRSA)*	14	14.7%
*Methicillin-Sensitive Staphylococcus aureus (MSSA)*	30	31.5%
*Coagulase-Negative Staphylococci (CoNS)*	15	15.7%
*Escherichia coli*	3	2.5%
*Streptococcus viridans*	3	2.5%
*Streptococcus caprae*	3	2.5%
*Staphylococcus epidermidis*	3	2.5%
*Pseudomonas aeruginosa*	3	2.5%
*Staphylococcus lugdunensis*	4	3.3%
*Enterobacter* spp., *Propionibacterium*, *Corynebacterium* spp. and *Streptococcus mitis*	4	0.8%

**Table 5:** Main features and outcomes of the studies included in the research.

RESEARCH STUDY	TREATMENT OPTIONS	INFECTION CURE OUTCOMES	ORTHOPEDIC CURE OUTCOMES	MEAN FOLLOW-UP
Kessler et al. (2014) [20]	21 DAIR, 10 Two-Stage, 3 Arthrodesis	67.6% infection-free	Successful limb salvage	38.5 months
Anastasio et al. (2023) [21]	6 DAIR, 7 Two-Stage, 4 Arthrodesis, 1 BKA	Not specified	94.7% limb salvage	Not specified
Pfahl et al. (2022) [22]	7 DAIR, 1 One-Stage, 8 Two-Stage, 2 Three-Stage	100% infection-free	Successful limb salvage	58.5 ± 57.8 months
Short et al. (2020) [23]	1 Two-Stage	Not specified	Revision	Not specified
Myerson et al. (2014) [24]	19 DAIR	84% infection free	4 revisions	13.4 months
Lachman et al. (2018) [25]	14 DAIR	46.1% infection-free Dee	46% limb salvage	24 months
Miller et al.(2017) [26]	1 Two-Stage	100% infection-free	Successful limb salvage	5 Years
Mulhern et al. (2016) [27]	1 Two-Stage	100% infection-free	Successful limb salvage	9 months
McCoy Jr. et al. (2012) [28]	3 Two-Stage	Not specified	94.7% limb salvage	7 months
Reuver et al. (2010) [19]	2 Arthrodesis	100% infection-free	Successful limb salvage	Not specified
Young et al. (2009) [29]	1 Two-Stage	100% infection-free	Successful limb salvage	16 months
Patton et al. (2012) [30]	4 DAIR, 1 One-Stage, 16 Two-Stage, 2 Spacer Arthroplasty, 6 BKA	65.5% infection-free	Not specified	Not specified
Kliushin et al. (2020) [31]	1 Two-Stage	100% infection-free	Successful limb salvage	12 months
Conti et al.(2022) [32]	11 Two-Stage	Not specified	66.3% limb salvage	14 months
Ferrao et al. (2012) [33]	6 Spacer Arthroplasty	90% infection-free	79.3% limb salvage	62 months

**Table 6:** Treatment modalities and complications.

TREATMENT OPTIONS	NUMBER OF PATIENTS	COMPLICATIONS (Infection Persistence)
DAIR	72 (48.8%)	33 (46.5%)
Revision One-Stage	2 (1.13%)	0
Revision Two-Stage	60 (34%)	12 (20%)
Revision Three-Stage	2 (1.13%)	1 (50%)
Primary Arthrodesis	14 (7.9%)	1 (7.14%)
Spacer Arthroplasty	8 (4.5%)	2 (25%)
Primary Amputation	7 (3.9%)	N/A

## Data Availability

All the data we analyzed and the tables we compiled are available for any clarification.
